# Disease Burden and Unmet Medical Needs in Patients with Ulcerative Colitis in Greece: A Cross-Sectional Patient Survey

**DOI:** 10.3390/medsci13030117

**Published:** 2025-08-08

**Authors:** George Gourzoulidis, Vasiliki-Rafaela Vakouftsi, George Mavridoglou, Marina Psarra, Charalampos Tzanetakos

**Affiliations:** 1 Health Through Evidence, 17456 Athens, Greece; m.psarra@hte.gr (M.P.); c.tzanetakos@hte.gr (C.T.); 2 Hellenic Society of Crohn’s Disease’s and Ulcerative Colitis’ Patients (HELLESCC), 11252 Athens, Greece; vasovak@yahoo.com; 3 Department of Accounting and Finance, School of Management, University of Peloponnese, 24100 Kalamata, Greece; ge.mavridoglou@go.uop.gr

**Keywords:** ulcerative colitis, disease burden, unmet medical need, patient-reported outcomes

## Abstract

**Background**: Ulcerative colitis (UC) requires life-long disease management. This study aimed to investigate the disease burden and unmet medical needs in UC patients in Greece. **Methods**: Between October 2023 and January 2024, adult UC patients who were members of the Hellenic Society of Crohn’s Disease and Ulcerative Colitis Patients (HELLESCC) completed a structured self-reported questionnaire. The survey questionnaire included sociodemographic characteristics, smoking habits, history of comorbidities, disease activity, disease characteristics, medications, and patient-reported outcomes (PROs; Short Inflammatory Bowel Disease Questionnaire [SIBDQ], Work Productivity and Activity Impairment [WPAI], Patient Health Questionnaire-9 [PHQ-9], treatment satisfaction, and treatment adherence). Univariate and multivariate logistic regression analyses were used to identify associated factors. **Results:** Datasets were obtained from 181 UC patients, of whom 48% were on advanced therapies (biological/small-molecule agents) and 54% had active disease. Around 74% reported impaired quality of life (QoL) (SIBDQ < 60), 25% work productivity loss, and 29% daily activity impairment. About 40% reported moderate to severe depressive symptoms (PHQ-9 ≥ 10). Reduced adherence and treatment dissatisfaction were reported by approximately one-third of patients. Female gender and disease activity were associated with moderately to severely impaired QoL, work productivity, and mental health. Interestingly, three out of four patients receiving advanced therapy reported moderately to severely impaired QoL and had increased odds of experiencing moderate to severe depression. **Conclusions**: The disease burden remains very high in UC, characterized by poor QoL and increased work impairment, depression, and disease activity among Greek patients. Marked treatment dissatisfaction and non-adherence were observed in approximately one-third of patients.

## 1. Introduction

Ulcerative colitis (UC) is a chronic inflammatory bowel disease (IBD) defined by relapsing–remitting colonic mucosa inflammation or a chronic active course [1]. In Greece, the incidence of UC is estimated at 5.6–8.9 new cases per 100,000 inhabitants per year [2]. UC patients often experience distressing, unpredictable gastrointestinal symptoms, comorbidities and treatment-related adverse events [3]. Disease burden has a wide impact on other aspects of patients’ lives, including employment, work efficiency, and social interactions [4,5]. These challenges may lead to an increased score of depression as well as a worse health-related quality of life (HRQoL) [6].

To fully address the burden of UC, it is essential to consider patient-reported outcomes (PROs) in addition to clinical outcomes, such as symptom control and remission. PROs provide critical insights into the impact of UC on patients’ quality of life (QoL), work productivity, and mental health [7,8,9,10]. Acknowledged as important endpoints for the treatment of IBD [11], PROs have been determined to be therapeutic targets by IBD experts [12,13,14]. A number of disease-specific and generic questionnaires have been developed and validated for the assessment of PROs in UC [15]. More specifically, the Short Inflammatory Bowel Disease Questionnaire (SIBDQ) has been widely employed to measure disease-specific QoL related to physical, emotional, and social aspects [7,16,17,18]. The burden of UC on work productivity has been studied with the Work Productivity and Activity Impairment (WPAI) questionnaire, including measures of absenteeism, presenteeism, and activity impairment [10,19,20,21,22]. Depression symptoms have also been evaluated by the Patient Health Questionnaire-9 (PHQ-9) [23,24,25].

Healthcare professionals often underestimate the disease burden while they fail to recognize important patient concerns, including HRQoL and shared decision-making [26]. A recent Greek survey estimated that 78% of patients with UC found the disease mentally exhausting, even among those in remission [3]. Recognizing these challenges, initiatives such as STRIDE-II by the International Organization for the Study of Inflammatory Bowel Disease emphasize the restoration of HRQoL as a fundamental long-term treatment objective [14]. Similarly, the European Crohn’s and Colitis Organization (ECCO) highlights the importance of evaluating HRQoL and patient satisfaction to maintain optimal standards of care [13]. These insights underscore the critical need for a comprehensive, patient-centered approach to UC management.

Relevant real-world patient-reported data can offer valuable insights into the HRQoL, satisfaction, and needs of patients with UC. This evidence can help identify areas in disease management that need improvement to align better with patients’ expectations and enhance their overall care experience, ultimately aiming for optimal disease control. In Greece, there is currently limited knowledge about the disease burden and unmet medical needs in patients with UC.

To this end, the primary objective of the present study was to investigate the overall disease burden and unmet medical need of UC by administering a questionnaire on PROs to members of the Greek national IBD patients’ association. Relationships between demographic and disease-related factors and these PROs were also investigated.

## 2. Materials and Methods

### 2.1. Study Design and Population

Between October 2023 and January 2024, a cross-sectional survey with a structured questionnaire was conducted in collaboration with the Hellenic Society of Crohn’s Disease and Ulcerative Colitis Patients (HELLESCC). Eligible to participate were adult (age ≥ 18 years) patients with UC, and members of this Greek patient association. The questionnaire was sent as a link via email or phone to all prospective participants. The recruitment process was conducted by HELLESCC staff without collecting participants’ personal data. All participants were informed in advance about the purpose of the study and were asked to provide their consent before participating. Participation in the survey was entirely voluntary, and participants had the right to withdraw their consent at any time. All data collected were kept anonymous and confidential.

### 2.2. Questionnaire and Variables

The questionnaire was developed in Greek and variables of interest comprised sociodemographic characteristics, smoking habits, history of comorbidities, disease activity, disease characteristics, current UC medications, and PROs (SIBDQ, WPAI-UC, PHQ-9, treatment satisfaction, and treatment adherence). Sociodemographic characteristics included gender, age, residence, weight, height, family status, socioeconomic status, and education level. Disease characteristics included age at diagnosis, time from symptom onset to diagnosis, history of doctor visits and hospitalization during the last 12 months for UC-related issues, and history of UC surgery. Disease activity was measured using the Simple Clinical Colitis Activity Index (SCCAI) score [27] and classes were defined as follows: remission: 0–2; mild: 3–5; moderate: 6–11; and severe: >11 [28,29]. Pertaining to UC therapy, all prescription medications available at the time of the survey were recorded, based on the national therapeutic protocol for UC issued by the Greek Ministry of Health [30]. More specifically, advanced therapies such as tumor necrosis factor inhibitors (TNFi), integrin α4 inhibitors, interleukin-12/23 inhibitors (IL-12/23 i), and Janus kinase inhibitors (JAKi) as well as non-advanced therapies such as 5-aminosalicylic acids (5-ASAs), corticosteroids, and immunosuppressants were included [30].

Validated questionnaires were used for the assessment of the following PROs: QoL (the SIBDQ), work productivity (the WPAI) and psychological burden (the PHQ-9) [18,19,25]. The SIBDQ score ranges from 10 to 70 [18,22]. QoL is considered to be slightly, moderately and severely worsened for SIBDQ scores of 60–70, 45–59 and 10–44, respectively [7,18,31]. Therefore, in this study, a SIBDQ score < 60 was defined as moderately to severely impaired QoL and ≥60 as normal QoL. The WPAI score (0–100%) measures absenteeism (work time missed), presenteeism (impairment at work), work productivity loss (overall work impairment/absenteeism plus presenteeism), and activity impairment [19,22]. Currently, there are no established thresholds to categorize the severity of the WPAI score. Similar to Williet et al. [7], the following severity categorization was employed: mild [0–19%], moderate [20–49%], severe [≥50%]. PHQ-9 scores range from 0 to 27, with higher scores indicating more severe depressive symptoms. The cut-off point of 10 or greater corresponds to moderate to severe depressive symptoms, potentially indicating a clinically significant condition (cut-off scores: 0–4 = minimal or none; 5–9 = mild; 10–14 = moderate; 15–19 = moderately severe; 20–27 = severe) [23,24,25]. The present study utilized the validated Greek versions of the SIBDQ, WPAI-UC, and PHQ-9 questionnaires, all of which are freely accessible [32,33,34,35].

Treatment satisfaction was assessed using a study-specific, 5-point Likert scale question, which measured participants’ level of satisfaction with their current treatment (not at all; little; quite; a lot; very much satisfied). For dissatisfied patients (grouped responses of “not at all”, “little”, and “quite satisfied”), the reasons for dissatisfaction were also collected (due to side effects; I have frequent stools; I still have frequent flares; the frequency of doses; the cost of the medications; I experience more abdominal pain; fatigue is increasing; I do not like the mode of administration; I experience more urgency to go to the bathroom; other). Treatment adherence was also evaluated using a study-specific, 5-point Likert scale question (I follow my treatment regularly; there are few times I forget to/I do not take my treatment; sometimes I forget to/I do not take my treatment; many times, I forget to/I do not take my treatment; I never take my treatment). For patients who do not adhere to their treatment regularly, the reasons for non-adherence were also collected and options included: I feel that my symptoms are under control; mode of administration; frequent drug doses; the drug is not effective; fear of side effects; other reasons.

### 2.3. Statistical Analysis

Categorical variables were summarized by frequencies (n) and percentages (%) and continuous variables with means and standard deviations (SDs). Sociodemographic variables, clinical variables, and PROs (SIBDQ, WPAI-UC, PHQ-9, treatment satisfaction, and treatment adherence) were described by the type of treatment [advanced or non-advanced]. The association of type of treatment with sociodemographic and clinical factors was investigated with the Pearson’s χ^2^ test for categorical variables and with the Mann–Whitney test for continuous variables. The mean or proportional differences between advanced and non-advanced treatment groups and their corresponding 95% confidence intervals (CIs) were also calculated. CIs enhance clinical interpretation by providing a plausible range of values in actual units of measurement, along with the direction and strength of the effect [36]. It is important to note that the outcome comparisons are cross-sectional and should not be interpreted as differences in response to treatment. Relationships between PROs and disease activity were quantified using Spearman’s correlation coefficient (SCC). Sociodemographic and clinical factors associated with PROs were analyzed at both bivariate and multivariate levels using logistic regression. Factors with a *p*-value < 0.15 in bivariate analyses were included in a multivariate logistic regression model with stepwise selection. Effect sizes are presented as odds ratios (ORs) with 95% CIs. Statistical significance was defined as *p* < 0.05. Data cleaning, data manipulation and data analysis were conducted using the statistical software IBM SPSS Statistics 29.0.

## 3. Results

### 3.1. Participants’ Characteristics

The questionnaire document was sent to 463 UC patients and returned by 197 [participation rate: 42.5%]. The survey required approximately 20 min to complete. A total of 181 patients were included in the final analysis (16 respondents who were not receiving any UC medication were excluded) (Table 1). The mean age [SD] was 42 years [11.0], and 46.4% were male. Two in ten (19.9%) patients were current smokers. The mean age at diagnosis was 30.7 years [10.7] and the mean disease duration was 11.4 years [8.2]. The mean time from symptom onset to diagnosis was 8.6 months [12.0]. During the last 12 months, the mean number of visits to doctors was 3.7 [9.5] and 11.1% of patients had at least one hospitalization. A total of 9 (6.3%) patients had a history of surgery and 82 (57.3%) reported at least one comorbidity (Supplementary Table S1).

Most patients (46.4%) were in remission, 34.8% had mild disease, and 18.8% had moderate to severe disease. Of these, 47.5% received advanced therapies (alone or in combination with non-advanced therapies) and 52.5% non-advanced (5-ASA, corticosteroids, immunosuppressants, antibiotics). Among biological agents, 53.5% were on TNF inhibitors, 20.9% on integrin inhibitors, 15.1% on interleukin-12/23 inhibitors, and 10.5% on JAK inhibitors.

The group of advanced therapies had a lower paid employment rate compared to the non-advanced group (56.9% vs. 80.2%, *p* = 0.002) and longer delays from symptom onset to diagnosis (11.5 vs. 6.0 months, *p* = 0.001). Patients who were receiving advanced therapies had almost three times more gastroenterologist visits (5.8 vs. 2.0, *p* = 0.015) and hospital admissions (18.8% vs. 5%, *p* = 0.009). Higher disease activity (moderate to severe: 27.9% vs. 10.5%, *p* = 0.003) was reported in advanced treatment patients.

### 3.2. Patient-Reported Outcomes

The data on PROs are summarized in Table 2. The mean SIBDQ score was 49.2 [13.6]. Moderately to severely impaired QoL [SIBDQ < 60] was reported by 74.2% of participants. Although rates in both treatment groups were similar, the majority of patients reported worsened QoL (76.4% vs. 72.4%, *p* = 0.568).

Of 159 participants who filled out the WPAI questionnaire, 116 (73.0%) were employed and thus able to respond. The treatment group receiving advanced therapy had a lower proportion of employed patients compared to those receiving non-advanced therapy (61.1% vs. 82.8%, *p* = 0.002). UC had a relatively small impact on absenteeism (mean [SD]: 7% [18.9%]); however, its effects on presenteeism (20.7% [25.3%]), work productivity loss (25.1% [29.5%]), and activity impairment (29.4% [29.8%]) were more pronounced. Moderate to severe absenteeism, presenteeism, work productivity and activity impairment were reported by 10.8%, 41.4%, 43.1% and 52.3% of patients, respectively. Notably, higher rates of moderate to severe absenteeism (20.5% vs. 4.8%, *p* = 0.013) and daily activity impairment (65.7% vs. 41.5%, *p* = 0.003) were observed in the advanced therapy group.

Two in five (39.6%) patients reported depression. The mean PHQ-9 score was 8.7 [6.9], which corresponds to mild symptoms. Half of patients (50.7%) on advanced therapies had moderate to severe depression [PHQ-9 ≥10] compared to 30.5% of those on non-advanced therapy (*p* = 0.012).

In total, 29.4% were ‘not at all,’ ‘a little,’ or ‘quite’ satisfied with their treatment. Dissatisfaction was higher in the advanced therapy group (33.7%), with ‘frequent flares’ being the leading cause (16.9%), followed by ‘increasing fatigue’ (14.5%) (Supplementary Table S2). Almost one-third (33.9%) of the population reported reduced adherence. Non-adherence was greater in the non-advanced therapy group (*p* = 0.01), while it was observed in approximately one-fourth of advanced receivers (reasons for non-adherence are provided in Supplementary Table S3).

### 3.3. Univariate and Multivariate Analysis

Univariate and multivariate logistic regressions were used to identify factors associated with moderately to severely impaired QoL [SIBDQ < 60], moderate to severe overall work impairment [WPAI ≥ 20%], moderate to severe depressive symptoms [PHQ-9 ≥ 10], treatment satisfaction, and treatment adherence (Table 3, Table 4 and Table 5).

In a multivariate analysis, the risk of moderately to severely impaired QoL was significantly higher in women (OR: 4.03, [95%CI: 1.65–9.83], *p* = 0.002). Patients with active disease had nearly twelve times higher odds of impaired QoL compared to those in remission (11.98, [4.23–33.88], *p* < 0.001). For moderate to severe overall work impairment, the odds were also significantly higher in women (3.45, [1.30–9.11], *p* = 0.013) and current smokers (4.37, [1.26–15.12], *p* = 0.020). Patients with active disease were nearly seven times more likely to have work impairment compared to those in remission (7.22, [2.75–18.97], *p* < 0.001).

Multivariate analysis also confirmed gender, disease severity, and type of treatment as factors independently associated with depression. More specifically, factors significantly associated with higher moderate to severe depressive symptoms included being female (3.86, [1.69–8.82], *p* = 0.001), having active disease (6.08, [2.66–13.91], *p* < 0.001), and receiving advanced treatments (2.45, [1.09–5.50], *p* = 0.029).

Pertaining to treatment satisfaction, multivariate analysis demonstrated that patients with active disease were significantly less likely to report treatment satisfaction compared to those in remission (0.13, [0.05–0.34], *p* < 0.001) (Supplementary Table S4). Interestingly, patients with one or more comorbidities had significantly higher odds of treatment satisfaction compared to those without comorbidities (2.83, [1.23–6.52], *p* = 0.014). In terms of treatment adherence, women had significantly lower odds of adhering to treatment compared to men (0.39, [0.20–0.79], *p* = 0.008) (Supplementary Table S5). Patients receiving advanced treatment were also two times more likely to adhere to treatment compared to those on non-advanced treatment (2.33, [1.19–4.58], *p* = 0.014).

### 3.4. Correlations Between PROs and Disease Activity

**Figure 1:**
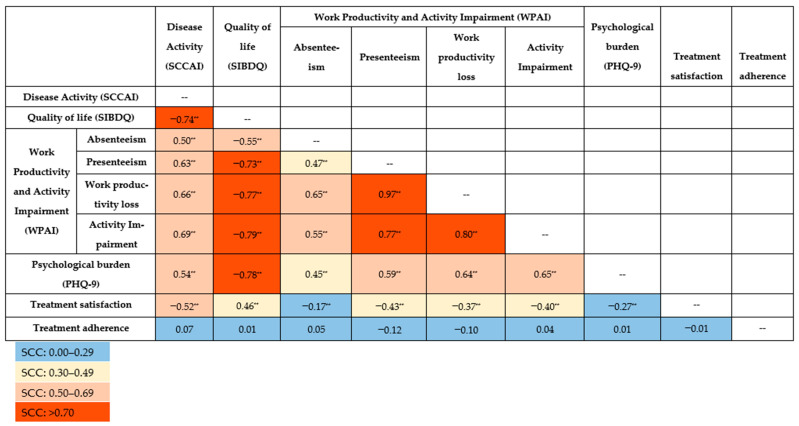
Spearman’s correlation coefficient (SCC) for patient-reported outcomes and disease activity. ** Correlation is significant at the 0.001 level (2-tailed). PHQ-9: Patient Health Questionnaire-9; SCCAI: Simple Clinical Colitis Activity Index; SIBDQ: Short Inflammatory Bowel Disease Questionnaire; WPAI: Work Productivity and Activity Impairment questionnaire.

All the SCCs between PROs and disease activity were statistically significant (*p* < 0.001), except for treatment adherence (Figure 1). Notably, very strong correlations were observed for presenteeism on the one hand and work productivity loss (SCC [95% CI]: 0.97 [0.96; 0.98]) and activity impairment (0.77 [0.68; 0.84]) on the other. Work productivity loss was also very strongly correlated with activity impairment (0.80 [0.72; 0.83]).

Decreased QoL (SIBDQ) was correlated with increased work and activity impairment: absenteeism (−0.55 [−0.67; −0.39]), presenteeism (−0.73 [−0.81; −0.62]), work productivity loss (−0.77 [−0.84; −0.67]), and activity impairment (−0.79 [−0.84; −0.72]). A very strong correlation was revealed between QoL and psychological burden (PHQ-9) (−0.78 [−0.84; −0.71]). Poorer QoL was moderately associated with lower treatment satisfaction (0.46 [0.32; 0.37]).

Disease activity (SCCAI) was inversely correlated with QoL (−0.74 [−0.80; −0.65]) and treatment satisfaction (−0.52 [−0.63; −0.41]). Additionally, disease activity was positively correlated with absenteeism (0.50 [0.34; 0.64]), presenteeism (0.63 [0.49; 0.74]), work productivity loss (0.66 [0.53; 0.76]), activity impairment (0.69 [0.60; 0.77]) and psychological burden (0.54 [0.41; 0.64]), indicating that greater disease activity contributes to increased impairment in work and daily activities.

The weakest but still significant correlations were associated with treatment satisfaction and absenteeism (−0.17 [−0.36; −0.09]) as well as psychological burden (−0.27 [−0.42; −0.11]). Correlations between treatment satisfaction on one side and work productivity loss (−0.37 [−0.53; −0.19]) and activity impairment (−0.40 [−0.53; −0.25]) on the other were moderate. Absenteeism was moderately correlated with psychological burden (0.45 [0.27; 0.59]). No correlations were observed between treatment adherence and other PROs or disease activity.

## 4. Discussion

Assessing the disease burden of UC is essential for enhancing and guiding potential treatment adjustments. Patient experience assessments have become a key component of nearly all recent large-scale randomized controlled trials in the field of IBD [37,38]. Complementing this, real-world PROs offer valuable additional insights into patients’ QoL, functional status, mental health, and treatment experiences, providing a more comprehensive, patient-centered perspective on the burden of illness in routine clinical practice. Hence, this study aimed to examine the impact of UC as reflected by PROs and to identify the unmet medical needs in a Greek real-world setting, where existing knowledge is limited (Supplementary Figure S1).

The mean SIBDQ score of 49.2 in this study reveals a diminished QoL, with 74% of patients reporting moderate to severe impairment. This is aligned with earlier studies where UC patients had a lower QoL due to disease symptoms and flares [7,18]. Notably, 76% of participants in advanced therapy reported moderately to severely reduced QoL, underlining the severity and the complexity of the disease. Consistent with previous studies [39], QoL was found to be more impaired in women, although this was not supported by earlier Greek research [40,41]. Multivariate analysis also confirmed that disease activity is negatively associated with QoL, aligning closely with other studies [7,9,40,42].

The negative impact of UC on productivity and daily activities was substantial. Absenteeism, presenteeism, work productivity loss, and activity impairment were reported as moderate to severe in approximately 11%, 41%, 43% and 52% of participants. These results are similar to those of the French BIRD study [7]. In the advanced therapy group, employment rates were lower, while absenteeism and activity impairment rates were higher, reflecting their greater disease severity and functional limitations [43]. Nonetheless, the type of treatment was not associated with overall work impairment in the multivariate setting. The negative impact of UC on productivity was also shown in an earlier Greek survey by Viazis et al. (2022) [3], where it was revealed that 88% of employed UC patients had at least one missed workday, 79% had missed work for treatment or medical appointments, and 81% felt that they would be more successful without the disease. Similar to other studies [43], disease activity was a predictor of moderate to severe work impairment. Consistent with previous studies [7], female gender was also a predictor of significant productivity. Smokers were also more likely to experience moderate to severe productivity loss as smoking exacerbates disease activity, impairs treatment effectiveness, and contributes to increased fatigue and psychological stress [44,45,46].

Due to the chronic nature of UC and its impact on daily life, patients experience higher rates of mental symptoms than the general population [47,48]. A meta-analysis reported depression and anxiety prevalence at 23% and 32.6%, respectively, in UC patients [49]. Consistent with the present study, women and patients with active disease were more likely to present moderate to severe depressive issues [49]. Additionally, patients receiving advanced therapy were more likely to have moderate to severe depression compared to those receiving non-advanced therapy. However, this likely reflects their higher disease burden.

Treatment dissatisfaction remained a concern for nearly 29% of all participants and one-third of those in the advanced treatment group. Compared to previous relevant research [50,51,52], treatment dissatisfaction was higher for patients on advanced treatment and lower for patients on non-advanced treatment in our study. Multivariate analysis confirmed that patients with active disease were 87% less likely to be satisfied with their treatment than patients in remission, emphasizing the significant impact of disease symptoms on treatment perceptions [53]. Interestingly, it was found that patients with one or more comorbidities had higher odds of being satisfied with their treatment. While this finding may seem counterintuitive, patients dealing with multiple health conditions may have lower expectations and place greater value on any improvement or stability in their health, leading to a more favorable perception of treatment effectiveness. It is important to emphasize that in this study, treatment satisfaction and dissatisfaction were used as general terms without consideration of specific criteria, similar to the approach taken for adherence and non-adherence below.

Treatment non-adherence is also a common problem among chronic diseases, including IBD, averaging 50% in developed countries [54]. The rate of 33.9% among these study participants is within the range of findings in other IBD populations [55,56]. Adherence was significantly higher in the advanced therapy group, suggesting greater engagement than those on non-advanced treatments. Consistent with previous research on IBDs [57,58], female gender was associated with lower odds of treatment adherence. Higher levels of psychological distress in women with IBD have been shown to negatively impact adherence to treatment regimens [39], which aligns with the findings of our study. Frequent flares, fatigue, and side effects, which were reported here as leading causes of dissatisfaction, are consistent with earlier reports noting that symptom persistence and side effects are major contributors to non-adherence and dissatisfaction [55,59]. These findings highlight the importance of patient-centered care and shared decision-making to optimize therapeutic outcomes.

The correlations between PROs and disease activity highlight the following interconnections: (i) poor QoL is associated with high productivity loss and high levels of depression; (ii) high disease activity is associated with poor QoL, low work productivity, and high levels of depression; (iii) patients with productivity loss are more likely to be depressed; and (iv) low treatment satisfaction is associated with high disease activity, poor QoL, and high productivity loss. Ghosh et al. observed a strong correlation between QoL (SIBDQ), disease activity (p-SCCAI) and depressive symptoms (PHQ-9) in patients with recent-onset UC [60]. Presenteeism, activity impairment, and work productivity loss were moderately correlated with disease activity and depressive symptoms [60]. Supporting the present findings, the BIRD study also demonstrated that poor QoL was strongly associated with greater productivity loss and higher rates of depression and anxiety [7]. A recent real-world study supported the correlation between higher disease activity and lower treatment satisfaction [53]. More specifically, Burisch et al. found that 71.7% of UC patients not in remission were dissatisfied with their treatment, compared to just 19.8% of those in remission [53]. IBD symptoms were significantly associated with reduced HRQoL, which contributed to a substantial proportion of patients not being satisfied with their treatment. To the best of our knowledge, the present study is the first Greek study to assess the correlations between PROs in UC.

This study has several strengths. First, it assessed various dimensions of PROs in UC, providing valuable insights into the disease burden within the Greek population. Second, multiple validated and non-validated tools were included. Third, real-world experiences of UC patients were included, offering data that are directly applicable to clinical practice and patient-centered care strategies. Female gender and disease activity emerged as the main factors associated with almost all PROs. Importantly, a pronounced proportion of UC patients receiving biologic therapies still experienced high disease burden, with poor QoL as well as increased work impairment, depression, and disease activity, highlighting the persistent unmet needs in these patients. Along with the considerable treatment dissatisfaction and non-adherence rates, there is an increasing need to incorporate assessments of PROs into treatment decision-making and estimates of health outcomes.

Several limitations must be acknowledged. As this was a cross-sectional survey, the ability to infer causality between PROs and other factors is intrinsically limited. Further, the study population was restricted to adult UC patients who are members of the HELLESCC, which may introduce selection bias. Nevertheless, HELLESCC is the official and only national IBD patient association in the country and also a member of the European Federation of Crohn’s & Ulcerative Colitis Associations (EFCCA). Self-reporting may also introduce interpretation bias since all patients self-completed the questionnaires. In the same direction, there is a lack of objective markers of disease activity such as endoscopy or disease markers using labs/fecal laboratory values. Recall bias or reporting bias may have also influenced responses. Another limitation is that treatment satisfaction and adherence were assessed using study-specific questionnaires. This may set limits to their accuracy and reliability, yet there are no “one-way” tools at present [55,61,62]. Despite their limitations, these tools provided important insights into the patient experience, which is vital for evidence-based care. Finally, while the questionnaire incorporated validated tools to assess core patient-reported outcomes, it was not developed based on a formal framework or model. This approach is consistent with similar PRO studies in the field [7,8,63,64]; however, future research aiming to investigate determinants or support intervention design may benefit from the application of such frameworks.

## 5. Conclusions

In conclusion, this cross-sectional survey highlights the substantial disease burden in UC, characterized by poor QoL, increased work impairment, high rates of depression, and elevated disease activity among Greek patients. Treatment dissatisfaction and non-adherence were also observed in approximately one-third of patients. Consistent with the literature, these findings confirm the persistent unmet medical needs in UC from a patient perspective. Close monitoring of patients in clinical practice, along with a thorough assessment of PROs and a shared decision-making model between patients and clinicians, could be the most effective strategy for reducing disease burden within the current therapeutic options.

## Figures and Tables

**Table 1:** Characteristics of the study population.

	Total (n = 181)	Advanced Therapies ^a^ (n = 86)	Non-Advanced Therapies ^b^ (n = 95)	Difference (95% CI) ^c^	*p*-Value ^d^
**Age, years**					
Mean [SD]	42.0 [11.0]	41.9 [11.2]	42.2 [10.9]	−0.32 (−3.56–2.93)	0.725
**Gender, n (%)**					
Male	84 (46.4%)	40 (46.5%)	44 (46.3%)	0.2% (−0.14–0.14)	0.979
**BMI, n (%)**					
Underweight (<18.5)	4 (2.2%)	1 (1.2%)	3 (3.2%)	--	0.148
Normal (18.5–25)	81 (44.8%)	33 (38.4%)	48 (50.5%)	−12.2% (−26.1–2.4%)	
Overweight (25–30)	61 (33.7%)	32 (37.2%)	29 (30.5%)	6.7% (−7.0–20.1%)	
Obese (≥30)	35 (19.3%)	20 (23.3%)	15 (15.8%)	7.5% (−4.2–18.9%)	
**Residence, n (%)**					
Urban area (>10,000 residents)	150 (82.9%)	71 (82.6%)	79 (83.2%)	−0.6% (−11.7–10.4%)	0.915
**Family status, n (%)**	n = 146	n = 65	n = 81		
Married	91 (62.3%)	36 (55.4%)	55 (67.9%)	−12.5% (−27.6–3.2%)	0.121
**Socioeconomic status, n (%)**	n = 146	n = 65	n = 81		
In paid employment §	102 (69.9%)	37 (56.9%)	65 (80.2%)	−23.3% (−37.5–−8.1%)	0.002
**Education level, n (%)**	n = 146	n = 65	n = 81		
Bachelor’s degree or more	94 (64.4%)	42 (64.6%)	52 (64.2%)	0.4 (−15.1–15.8%)	0.958
**Smoker, n (%)**	181	86	95		
Current smoker	36 (19.9%)	18 (20.9%)	18 (18.9%)	2.0% (−9.7–13.7%)	0.739
Former smoker	63 (34.8%)	30 (34.9%)	33 (34.7%)	0.1% (−13.6–14.0%)	0.983
Never smoker	82 (45.3%)	38 (44.2%)	44 (46.3%)	−2.1% (16.4–12.3%)	0.774
**Age at diagnosis, years**	181	86	95		
Age at diagnosis, mean [SD]	30.7 [10.7]	30.4 [11.6]	30.9 [9.8]	−0.5 (−3.6–2.7)	0.770
**Disease duration, years**	181	86	95		
Disease duration, mean [SD]	11.4 [8.2]	11.5 [6.9]	11.3 [9.2]	0.15 (−2.3–2.3)	0.902
**Time from symptom onset to diagnosis, months**	181	86	95		
Time from onset, mean [SD]	8.6 [12.0]	11.5 [16.1]	6.0 [3.0–13.0]	5.6 (2.2–9.0)	0.001
**Surgery, n (%)**	144	64	80		
Surgery	9 (6.3%)	5 (7.8%)	4 (5.0%)	2.8% (−7.0–11.6%)	0.488
**Gastroenterologist visits in the past 12 months**	144	64	80		
Number of visits, mean [SD]	3.7 [9.5]	5.8 [13.7]	2.0 [2.4]	3.9 (0.8–7.0)	0.015
**Hospitalization in the past 12 months, n (%)**	144	64	80		
Hospitalization	16 (11.1%)	12 (18.8%)	4 (5.0%)	13.7% (2.7–24.5%)	0.009
**Comorbidities, n (%)**	143	63	80		
One or more	82 (57.3%)	40 (63.5%)	42 (52.5%)	11, 0% (−5, 3–26, 6%)	0187
Disease Activity *, n (%)	181	86	95		
Remission	84 (46.4%)	36 (41.9%)	48 (50.5%)	−8.7% (−22.8–5.9%)	0.243
Mild	63 (34.8%)	26 (30.2%)	37 (38.9%)	−8.7% (−22, 8–5.9%)	0.219
Moderate to severe	34 (18.8%)	24 (27.9%)	10 (10.5%)	17.4% (5.7–28.4%)	0.003
Moderate	32 (17.7%)	23 (26.7%)	9 (9.5%)	17.3% (5.9–28.1%)	0.002
Severe	2 (1.1%)	1 (1.2%)	1 (1.1%)	--	--
**Ongoing treatment**					
***Non-advanced therapies, n (%)***	181	86	95		
5-ASA	144 (79.5%)	51 (59.3%)	93 (97.9%)	--	
Corticosteroids	21 (11.6%)	13 (15.1%)	8 (8.4%)	--	
Immunosuppressants	32 (17.6%)	20 (23.3%)	12 (12.6%)	--	
Antibiotics	6 (3.3%)	3 (3.5%)	3 (3.2%)	--	
***Advanced therapies, n (%)***	181	86			
TNF inhibitors	46 (25.4%)	46 (53.5%)	--	--	
Integrin α4 inhibitors	18 (9.9%)	18 (20.9%)	--	--	
Interleukin-12/23 inhibitors	13 (7.2%)	13 (15.1%)	--	--	
JAK inhibitors	9 (5.0%)	9 (10.5%)	--	--	

* SCCAI [remission: <2; mild: 3 to 5; moderate: 6 to 11; severe: >11]. § Full- or part-time employment or self-employed. ^a^ Advanced therapies: tumor necrosis factor inhibitors, integrin α4 inhibitors, interleukin-12/23 inhibitors, and Janus kinase inhibitors. ^b^ Non-advanced therapies: 5-aminosalicylic acids, corticosteroids, immunosuppressants, and antibiotics. ^c^ Unadjusted mean difference (95% CI) between groups for continuous variables and difference in percentage points (95% CI) between groups for categorical variables. ^d^ Pearson’s χ^2^ test or Mann–Whitney test. Abbreviations: 5-ASA: 5-aminosalicylic acid; BMI: body mass index; IQR: interquartile range; JAK: Janus kinase; TNF: tumor necrosis factor.

**Table 2:** Patient-reported outcomes in the study population.

PROs	Total (n = 181)	Advanced Therapies ^a^ (n = 86)	Non-Advanced Therapies ^b^ (n = 95)	Difference (95% CI) ^c^	*p*-Value ^d^
**Quality of life**					
SIBDQ	n = 159	n = 72	n = 87		
Mean [SD]	49.2 [13.6]	47.4 [14.6]	50.7 [12.6]	−3.3 (−7.6–0.9)	0.124
Moderate to severe impact [<60] n (%)	118 (74.2%)	55 (76.4%)	63 (72.4%)	4.0% (−9.8–17.3%)	0.568
**Productivity loss**					
WPAI					
Absenteeism	n = 102	n = 39	n = 63		
Mean [SD]	7% [18.9%]	12.4% [27.7%]	3.6% [9.2%]	8.7% (−0.5–17.9%)	0.064
Moderate to severe impact [≥20%], n (%)	11 (10.8%)	8 (20.5%)	3 (4.8%)	15.8% (1.8–29.7%)	0.013
Presenteeism	n = 99	n = 36	n = 63		
Mean [SD]	20.7% [25.3%]	23.9% [28,5%]	18.9% [23.4%]	5% (−6.2–16.2%)	0.056
Moderate to severe impact [≥20%], n (%)	41 (41.4%)	15 (41.7%)	26 (41.3%)	0.4% (−19.2–20.3%)	0.969
Work productivity loss	n = 102	n = 39	n = 63		
Mean [SD]	25.1% [29.5%]	32.2% [35.5%]	20.8% [24.2%]	11.4% (−1.6–24.3%)	0.084
Moderate to severe impact [≥20%], n (%)	44 (43.1%)	18 (46.2%)	26 (41.3%)	4.9% (−14.6–24.2)	0.628
Activity impairment	n = 149	n = 67	n = 82		
Mean [SD]	29.4% [29.8%]	39.4% [33.2%]	21.2% [24.1%]	18.2% (8.6–27.8%)	<0.001
Moderate to severe impact [≥20%], n (%)	78 (52.3%)	44 (65.7%)	34 (41.5%)	24.2 (8.1–39.0%)	0.003
**Psychological burden**					
PHQ-9	n = 149	n = 67	n = 82		
Mean [SD]	8.7 [6.9]	10.4 [7.9]	7.3 [5.7]	3.04 (0.75–5.33)	0.010
Moderate to severe impact [≥10], n (%)	59 (39.6%)	34 (50.7%)	25 (30.5%)	20.3% (4.4–34.9%)	0.012
**Treatment satisfaction**	n = 177	n = 83	n = 94		
Yes, n (%) ^¥^	125 (70.6%)	55 (66.3%)	70 (74.5%)	−8.2% (−21.4–5.2)	0.232
**Treatment adherence**	n = 177	n = 83	n = 94		
Yes, n (%) ^§^	177 (66.1%)	63 (75.9%)	54 (57.4%)	18.5% (4.5–31.5%)	0.010

^¥^ Responses for “a lot/very much satisfied”. ^§^ Responses for “I follow my treatment regularly”. ^a^ Advanced therapies: tumor necrosis factor inhibitors, integrin α4 inhibitors, interleukin-12/23 inhibitors, and Janus kinase inhibitors. ^b^ Non-advanced therapies: 5-aminosalicylic acids, corticosteroids, immunosuppressants, and antibiotics. ^c^ Unadjusted mean difference (95% CI) between groups for continuous variables and difference in percentage points (95% CI) between groups for categorical variables. ^d^ Pearson’s χ^2^ test or Mann–Whitney test. Abbreviations: IQR: interquartile range; PHQ-9: Patient Health Questionnaire-9; PROs: patient-reported outcomes; SIBDQ: Short Inflammatory Bowel Disease Questionnaire.

**Table 3:** Factors associated with moderately to severely [SIBDQ < 60] impaired QoL: univariate and multivariate logistic regression analyses.

SIBDQ	Univariate Analysis		Multivariate Analysis	
	OR [95% CI]	*p*-Value	OR [95% CI]	*p*-Value
**Gender**				
Male	Ref		Ref	
Female	4.05 [1.92–8.56]	<0.001	4.03 [1.65–9.83]	0.002
**Age**				
<50 years	Ref		Ref	
50 years or more	0.42 [0.20–0.88]	0.023	0.39 [0.12–1.24]	0.111
**Employment status**				
In paid employment	Ref			
Without paid employment	1.47 [0.63–3.45]	0.374		
**BMI**				
Underweight and normal	Ref			
Overweight and obese	0.80 [0.39–1.62]	0.533		
**Smoking status**				
Never smoker	Ref		Ref	
Former smoker	0.66 [0.31–1.40]	0.276	0.59 [0.20–1.72]	0.336
Current smoker	2.33 [0.72–7.53]	0.149	2.49 [0.63–9.82]	0.191
Disease activity *				
Inactive	Ref			
Active	10.96 [4.26–28.19]	<0.001	11.98 [4.23–33.88]	<0.001
**Age at diagnosis**				
0–30 years	Ref			
>30 years	1.37 [0.67–2.82]	0.39		
**Disease duration**				
<10 years	Ref			
10–19 years	0.75 [0.34–1.67]	0.481		
20 years or more	0.61 [0.23–1.59]	0.310		
**Surgery**	N/A			
**Ongoing treatments ^ⱡ^**				
Non-advanced	Ref			
Advanced	1.23 [0.60–2.53]	0.569		
**Comorbidities**				
None	Ref			
One or more	1.20 [0.56–2.54]	0.639		

* Inactive: Patients in remission. Active: patients with mild, moderate or severe disease activity. ^ⱡ^ Advanced therapies: tumor necrosis factor inhibitors, integrin α4 inhibitors, interleukin-12/23 inhibitors, and Janus kinase inhibitors. Non-advanced therapies: 5-aminosalicylic acids, corticosteroids, immunosuppressants, and antibiotics. Abbreviations: CI: confidence interval; BMI: body mass index; N/A: not applicable; OR: odds ratio; ref: reference value; SIBDQ: Short Inflammatory Bowel Disease Questionnaire.

**Table 4:** Factors associated with moderate to severe overall work impairment (WPAI ≥ 20%): univariate and multivariate logistic regression analyses.

WPAI	Univariate Analysis		Multivariate Analysis	
	OR [95% CI]	*p*-Value	OR [95% CI]	*p*-Value
**Gender**				
Male	Ref		Ref	
Female	2.94 [1.30–6.65]	0.010	3.45 [1.30–9.11]	0.013
**Age**				
<50 years	Ref			
50 years or more	0.70 [0.26–1.85]	0.470		
**BMI**				
Underweight and normal	Ref			
Overweight and obese	1.26 [0.57–2.76]	0.567		
**Smoking status**				
Never	Ref		Ref	
Former smoker	0.66 [0.25–1.74]	0.405	0.67 [0.22–2.09]	0.492
Current smoker	2.95 [1.02–8.55]	0.046	4.37 [1.26–15.12]	0.020
Disease activity *				
Inactive	Ref			
Active	6.43 [2.69–15.38]	<0.001	7.22 [2.75–18.97]	<0.001
**Age at diagnosis**				
0–30 years	Ref			
>30 years	0.94 [0.42–2.11]	0.871		
**Disease duration**				
<10 years	Ref			
10–19 years	0.96 [0.38–2.43]	0.922		
20 years or more	0.98 [0.36–2.65]	0.967		
**Surgery**	0.34 [0.04–3.14]	0.340		
**Ongoing treatments ^ⱡ^**				
Not advanced	Ref			
Advanced	1.22 [0.55–2.73]	0.629		
**Comorbidities**				
No	Ref			
One or more	1.01 [0.45–2.50]	0.984		

* Inactive: patients in remission. Active: patients with mild, moderate or severe disease activity. ^ⱡ^ Advanced therapies: tumor necrosis factor inhibitors, integrin α4 inhibitors, interleukin-12/23 inhibitors, and Janus kinase inhibitors. Non-advanced therapies: 5-aminosalicylic acids, corticosteroids, immunosuppressants, and antibiotics. Abbreviations: ΒΜΙ: body mass index; CI: confidence interval; OR: odds ratio; ref: reference value; WPAI: Work Productivity and Activity Impairment questionnaire.

**Table 5:** Factors associated with PHQ-9: univariate and multivariate logistic regression analyses.

PHQ-9	Univariate Analysis		Multivariate Analysis	
	OR [95% CI]	*p*-Value	OR [95% CI]	*p*-Value
**Gender**				
Male	Ref		Ref	
Female	4.02 [1.94–8.34]	<0.001	3.86 [1.69–8.82]	0.001
**Age**				
<50 years	Ref			
50 years or more	0.80 [0.38–1.67]	0.544		
**Employment status**				
In paid employment	Ref			
Without paid employment	1.67 [0.82–3.43]	0.159		
**BMI**				
Underweight and normal	Ref			
Overweight and obese	1.01 [0.54–1.95]	0.975		
**Smoking status**				
Never	Ref			
Former smoker	0.78 [0.37–1.64]	0.504		
Current smoker	1.27 [0.52–3.07]	0.598		
Disease activity *				
Inactive	Ref		Ref	
Active	6.11 [2.89–12.94]	<0.001	6.08 [2.66–13.91]	<0.001
**Age at diagnosis**				
0–30 years	Ref			
>30 years	0.86 [0.44–1.67]	0.650		
**Disease duration**				
<10 years	Ref			
10–19 years	0.82 [0.39–1.72]	0.594		
20 years or more	0.65 [0.26–1.63]	0.354		
**Surgery**	0.75 [0.18–3.13]	0.693		
**Ongoing treatments ^ⱡ^**				
Not advanced	Ref		Ref	
Advanced	2.35 [1.20–4.60]	0.013	2.45 [1.09–5.50]	0.029
**Comorbidities**				
No	Ref		Ref	
One or more	1.82 [0.91–3.64]	0.092	1.38 [0.61–3.09]	0.441

* Inactive: patients in remission. Active: patients with mild, moderate or severe disease activity. ^ⱡ^ Advanced therapies: tumor necrosis factor inhibitors, integrin α4 inhibitors, interleukin-12/23 inhibitors, and Janus kinase inhibitors. Non-advanced therapies: 5-aminosalicylic acids, corticosteroids, immunosuppressants, and antibiotics. Abbreviations: BMI: body mass index; CI: confidence interval; OR: odds ratio; ref: reference value; PHQ-9: Patient Health Questionnaire.

## Data Availability

All data generated or analyzed during this study are included in this published article/as Supplementary Files.

## Supplementary Materials

The following supporting information can be downloaded at: https://www.mdpi.com/article/10.3390/medsci13030117/s1. Table S1: Comorbidities stratified by treatment type; Table S2: Treatment satisfaction and reasons for dissatisfaction stratified by treatment type; Table S3: Treatment adherence and reasons for non-adherence stratified by treatment type; Table S4: Factors associated with treatment satisfaction†: univariate and multivariate logistic regression analyses; Table S5: Factors associated with treatment adherence†: univariate and multivariate logistic regression analyses; Figure S1: Overview of study outcomes.

## Abbreviations

The following abbreviations are used in this manuscript:

5-ASAs	5-Aminosalicylic Acids
CIs	Confidence Intervals
ECCO	European Crohn’s and Colitis Organization
EFCCA	European Federation of Crohn’s & Ulcerative Colitis Associations
HELLESCC	Hellenic Society of Crohn’s Disease and Ulcerative Colitis Patients
HRQoL	Health-Related Quality of Life
IBD	Inflammatory Bowel Disease
IL-12/23 i	Interleukin-12/23 Inhibitor
JAKi	Janus Kinase Inhibitor
OR	Odds Ratios
PHQ-9	Patient Health Questionnaire-9
PROs	Patient-Reported Outcomes
QoL	Quality of Life
SCC	Spearman’s Correlation Coefficient
SCCAI	Simple Clinical Colitis Activity Index
SD	Standard Deviations
SIBDQ	Short Inflammatory Bowel Disease Questionnaire
TNFi	Tumor Necrosis Factor Inhibitors
UC	Ulcerative Colitis
WPAI	Work Productivity and Activity Impairment
