# Supplementary material for: Disease Burden and Unmet Medical Needs in Patients with Ulcerative Colitis in Greece: A Cross-Sectional Patient Survey
Source: Med Sci (Basel). 2025 Aug 8;13(3):117. doi: 10.3390/medsci13030117 (PMC12371981; doi:10.3390/medsci13030117)
Supplement: Supplementary file 1 [file medsci-13-00117-s001.zip › medsci-3776475-supplementary.pdf]

Supplementary Materials

Supplementary Table S1. Comorbidities Stratified by Treatment Type

Comorbidities	Total (N=82)	Advanced Therapies [a] (N=40)	Non-advanced Therapies [b] (N=42)
COPD	2 (2.4%)	1 (2.5%)	1 (2.4%)
Arthritis	20 (24.4%)	15 (37.5%)	5 (11.9%)
Iron deficiency anaemia	14 (17.1%)	8 (20%)	6 (14.3%)
Hypothyroidism	12 (14.6%)	6 (15%)	6 (14.3%)
Skin allergies or other skin conditions	16 (19.5%)	8 (20%)	8 (19%)
Cancer	0 (0%)	0 (0%)	0 (0%)
Depression	18 (21.9%)	13 (32.5%)	5 (11.9%)
Diabetes	6 (7.3%)	5 (12.5%)	1 (2.4%)
Hypertension	16 (19.5%)	7 (17.5%)	9 (21.4%)
Cardiovascular disease	4 (4.9%)	4 (10%)	0 (0%)
Coronary artery disease	5 (6.1%)	3 (7.5%)	2 (4.8%)
Osteoporosis	11 (13.4%)	7 (17.5%)	4 (9.5%)
Migraine or severe headache	12 (14.6%)	6 (15%)	6 (14.3%)
Other	26 (31.7%)	12 (30%)	14 (33.3%)

[a] Advanced therapies: tumor necrosis factor inhibitors, integrin α4 inhibitors, interleukin-12/23 inhibitors, and Janus kinase inhibitors. [b] Non-advanced therapies: 5-aminosalicylic acids, corticosteroids, immunosuppressants, and antibiotics. Abbreviations: COPD: chronic obstructive pulmonary disease.

Supplementary Table S2. Treatment Satisfaction and Reasons of dissatisfaction Stratified by Treatment Type

	Total (N=177)	Advanced therapies [a] (N=83)	Non-advances Therapies [b] (N=94)
**Treatment satisfaction, n (%)**			
Not at all	3 (1.7%)	3 (3.6%)	0 (0%)
Little	16 (9%)	7 (8.4%)	9 (9.6%)
Quite	33 (18.6%)	18 (21.7%)	15 (16%)
A lot	47 (26.6%)	15 (18.1%)	32 (34%)
Very much	78 (44.1%)	40 (48.2%)	38 (40.4%)
**Reasons of dissatisfaction, n (%)**			
Side effects	12 (6.8%)	8 (9.6%)	4 (4.3%)
I have frequent stools	10 (5.6%)	8 (9.6%)	2 (2.1%)
I have frequent flares	28 (15.8%)	14 (16.9%)	14 (14.9%)

	Total (N=177)	Advanced therapies [a] (N=83)	Non-advances Therapies [b] (N=94)
The frequency of doses	6 (3.4%)	4 (4.8%)	2 (2.1%)
The cost of medications	7 (4%)	1 (1.2%)	6 (6.4%)
I experience more abdominal pain	9 (5.1%)	3 (3.6%)	6 (6.4%)
Fatigue is increasing	19 (10.7%)	12 (14.5%)	7 (7.4%)
I do not like the mode of administration	3 (1.7%)	3 (3.6%)	0 (0%)
I experience more urgency to go to the bathroom	6 (3.4%)	3 (3.6%)	3 (3.2%)
Other reason	7 (4%)	4 (4.8%)	3 (3.2%)

[a] Advanced therapies: tumor necrosis factor inhibitors, integrin α4 inhibitors, interleukin-12/23 inhibitors, and Janus kinase inhibitors. [b] Non-advanced therapies: 5-aminosalicylic acids, corticosteroids, immunosuppressants, and antibiotics.

Supplementary Table S3. Treatment Adherence and Reasons of Non-Adherence Stratified by Treatment Type

	All (N=177)	Advanced therapies [a] (N=83)	Non-advanced Therapies [b] (N=94)
**Treatment adherence, n (%)**			
I follow my treatment regularly	117 (66.1%)	63 (75.9%)	54 (57.4%)
There are few times I forget to/ I do not take my treatment	43 (24.3%)	15 (18.1%)	28 (29.8%)
Sometimes I forget to/ I do not take my treatment	9 (5.1%)	4 (4.8%)	5 (5.3%)
Many times, I forget to/ I do not take my treatment	7 (4%)	1 (1.2%)	6 (6.4%)
I never take my treatment	1 (0.6%)	0 (0%)	1 (1.1%)
**Reasons of non-adherence, n (%)**			
I feel that my symptoms are under control	27 (15.3%)	7 (8.4%)	20 (21.3%)
Mode of administration	5 (2.8%)	1 (1.2%)	4 (4.3%)
Frequent drug doses	8 (4.5%)	1 (1.2%)	7 (7.4%)
The drug is not effective	3 (1.7%)	1 (1.2%)	2 (2.1%)
Fear of side effects	1 (0.6%)	1 (1.2%)	0 (0%)
Other reason	24 (13.6%)	10 (12%)	14 (14.9%)

[a] Advanced therapies: tumor necrosis factor inhibitors, integrin α4 inhibitors, interleukin-12/23 inhibitors, and Janus kinase inhibitors. [b] Non-advanced therapies: 5-aminosalicylic acids, corticosteroids, immunosuppressants, and antibiotics.

Supplementary Table S4. Factors associated with treatment satisfaction†: univariate and multivariate logistic regressions analyses

Satisfaction	Univariate analysis		Multivariate analysis	
	OR [95% CI]	p-value	OR [95% CI]	p-value
**Gender**				
Male	Ref			
Female	0.99 [0.52 – 1.87]	0.962		
**Age**				
<50 years	Ref			
50 years or more	1.25 [0.59 – 2.65]	0.563		
**Employment status**				
In paid employment	Ref			
Without paid employment	0.95 [0.43 – 2.06]	0.891		
**BMI**				
Underweight and normal	Ref			
Overweight and obese	1.11 [0.58 – 2.11]	0.750		
**Smoking status**				
Never	Ref			
Former smoker	1.01 [0.49 – 2.09]	0.981		
Current smoker	0.84 [0.36 – 1.95]	0.841		
**Disease activity** *				
Inactive	Ref		Ref	
Active	0.16 [0.08 – 0.36]	<0.001	0.13 [0.05 – 0.34]	<0.001
**Age at diagnosis**				
0-30 years	Ref			
>30 years	0.74 [0.39 – 1.40]	0.349		
**Disease duration**				
<10 years	Ref			
10–19 years	0.90 [0.44 – 1.83]	0.764		
20 years or more	0.91 [0.36 – 2.28]	0.84		
**Surgery**	0.78 [0.19 – 3.29]	0.739		
**Ongoing treatments** ł				
Not advanced	Ref			
Advanced	0.67 [0.35 – 1.29]	0.233		
**Comorbidities**				
No	Ref		Ref	
One or more	1.87 [0.90 – 3.89]	0.094	2.83 [1.23 – 6.52]	0.014

†Logistic regression: the dependent variable of satisfaction was categorized as 0: "not at all/little/quite satisfied" and 1: "a lot/very much satisfied". * Inactive: Patients in remission. Active: Patients with mild, moderate or severe disease activity. ł Advanced therapies: tumor necrosis factor inhibitors, integrin α4 inhibitors, interleukin-12/23 inhibitors,

and Janus kinase inhibitors. Non-advanced therapies: 5-aminosalicylic acids, corticosteroids, immunosuppressants, and antibiotics. Abbreviations: BMI: body mass index; CI: confidence interval; OR: odds ratio; ref: reference value.

Supplementary Table S5. Factors associated with treatment adherence†: univariate and multivariate logistic regressions analyses

Adherence	Univariate analysis		Multivariate analysis	
	OR [95% CI]	p-value	OR [95% CI]	p-value
**Gender**				
Male	Ref		Ref	
Female	0.40 [0.23 – 0.69]	0.006	0.39 [0.20 – 0.79]	0.008
**Age**				
<50 years	Ref			
50 years or more	1.08 [0.59 – 1.96]	0.832		
**Employment status**				
In paid employment	Ref			
Without paid employment	1.54 [0.72 – 3.29]	0.267		
**BMI**				
Underweight and normal	Ref			
Overweight and obese	1.02 [0.55 – 1.89]	0.951		
**Smoking status**				
Never	Ref			
Former smoker	1.06 [0.53 – 2.11]	0.869		
Current smoker	1.54 [0.65 – 3.62]	0.327		
**Disease activity** *				
Inactive	Ref			
Active	1.06 [0.57 – 1.97]	0.867		
**Age at diagnosis**				
0-30 years	Ref			
>30 years	1.08 [0.58 – 2.01]	0.803		
**Disease duration**				
<10 years	Ref		Ref	
10–19 years	1.85 [0.91 – 3.75]	0.090	1.58 [0.75 – 3.43]	0.288
20 years or more	1.11 [0.47 – 2.63]	0.820	1.03 [0.41 – 2.62]	0.945
**Surgery**	5.01 [0.61 – 41.2]	0.234		
**Ongoing treatments** ǂ				
Not advanced	Ref		Ref	
Advanced	2.33 [1.22 – 4.46]	0.010	2.33 [1.19 – 4.58]	0.014
**Comorbidities**				
No	Ref			
One or more	1.18 [0.60 – 2.35]	0.626		

†Logistic regression: the dependent variable of adherence was categorized as 0: "I forget to/ I do not take my treatment (few/some/many times /always)" and 1: "I follow my treatment regularly". * Inactive: Patients in remission.

Active: Patients with mild, moderate or severe disease activity. [‡] Advanced therapies: tumor necrosis factor inhibitors, integrin α4 inhibitors, interleukin-12/23 inhibitors, and Janus kinase inhibitors. Non-advanced therapies: 5-aminosalicylic acids, corticosteroids, immunosuppressants, and antibiotics. Abbreviations: BMI: body mass index; CI: confidence interval; OR: odds ratio; ref: reference value.

Figure S1. Overview of study outcomes

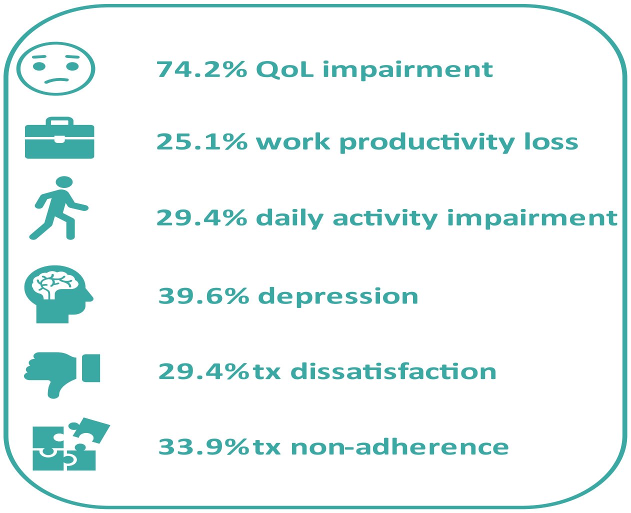

Abbreviations: QoL: quality of life; tx: treatment.
